# Efficacy of Kombucha Obtained from Green, Oolong, and Black Teas on Inhibition of Pathogenic Bacteria, Antioxidation, and Toxicity on Colorectal Cancer Cell Line

**DOI:** 10.3390/microorganisms7120700

**Published:** 2019-12-14

**Authors:** Thida Kaewkod, Sakunnee Bovonsombut, Yingmanee Tragoolpua

**Affiliations:** 1 Department of Biology, Faculty of Science, Chiang Mai University, Chiang Mai 50200, Thailand; tda007suju@gmail.com (T.K.); sakunnee.b@cmu.ac.th (S.B.); 2 The Graduate School, Chiang Mai University, Chiang Mai 50200, Thailand; 3 Center of Excellence in Bioresources for Agriculture, Industry, and Medicine, Department of Biology, Faculty of Science, Chiang Mai University, Chiang Mai 50200, Thailand

**Keywords:** antibacteria, antioxidation, Caco-2 cancer cells, kombucha tea, pathogenic bacteria

## Abstract

Kombucha tea is a refreshing beverage that is produced from the fermentation of tea leaves. In this study, kombucha tea was prepared using 1% green tea, oolong tea, and black tea, and 10% sucrose with acetic acid bacteria and yeast. The pH values of the kombucha tea were found to be in a range of 2.70–2.94 at 15 days of fermentation. The lowest pH value of 2.70 was recorded in the kombucha prepared from black tea. The total acidity of kombucha prepared from black tea was the highest by 16.75 g/L and it was still maintained after heat treatment by boiling and after autoclaved. Six organic acids: glucuronic, gluconic, D-saccharic acid 1,4-lactone, ascorbic, acetic, and succinic acid in kombucha tea were detected by HPLC with the optimization for organic acids detection using isocratic elution buffer with C18 conventional column. The highest level of organic acid was gluconic acid. Kombucha prepared from green tea revealed the highest phenolic content and antioxidation against DPPH radicals by 1.248 and 2.642 mg gallic acid/mL kombucha, respectively. Moreover, pathogenic enteric bacteria: *Escherichia coli*. *E. coli* O157:H7. *Shigella dysenteriae*, *Salmonella* Typhi, and *Vibrio cholera* were inhibited by kombucha and heat-denatured kombucha with diameter of the inhibition zones ranged from 15.0 ± 0.0–25.0 ± 0.0 mm. In addition, kombucha prepared from green tea and black tea demonstrated toxicity on Caco-2 colorectal cancer cells. Therefore, kombucha tea could be considered as a potential source of the antioxidation, inhibition of pathogenic enteric bacteria, and toxicity on colorectal cancer cells.

## 1. Introduction

Kombucha tea is a slightly acidic beverage that is produced from the fermentation of tea leaves (*Camellia sinensis*) and infusion with a consortium culture of acetic bacteria including *Acetobacter xylinum*, *A. xylinoides*, or *Bacterium gluconicum* and yeasts such as *Saccharomyces cerevisiae*, *S. ludwigii*, *Zygosaccharomyces bailii*, *Z. rouxii*, *Schizosaccharomyces pombe*, *Torulaspora delbrueckii*, *Brettanomyces bruxellensis*, *B. lambicus*, *B. custersii*, *Candida* sp., or *Pichia membranaefaciens* [1]. Normally, a traditional substrate used in kombucha fermentation is comprised of 10 g/L of black tea infusion that has been sweetened with.5–8% (*w*/*v*) sucrose. Notably, cellulose is produced during the fermentation by *A*. *xylinum* and appears as a thin film on top of the fermented tea where the cell mass of bacteria and yeast is attached. Yeast and bacteria in kombucha are involved in metabolic activities that utilize substrates in different pathways. Yeast cells hydrolyze sucrose into glucose and hydrolyze fructose using invertase enzymes. Moreover, ethanol is also produced and further utilized by acetic acid bacteria to generate organic acids and other substances such as acetic, gluconic acid, glucuronic acid, citric acid, lactic acid, malic acid, succinic acid, saccharic acid, pyruvic acid, sugars, vitamins, and amino acids [2]. Thus, the pH value of kombucha is known to decrease during the process of fermentation due to the production of organic acids. Kombucha beverages also contain other substances such as phenolic compounds in a quantity of about 30% (*w*/*w*) of the dry mass of the tea leaves [3]. These substances vary considerably depending on the variety of tea and the processing procedures. Moreover, kombucha fermentation is dependent upon the source of kombucha culture (tea fungus), which is affected by a variety of properties for each kombucha beverage. When compared to unfermented tea, the enhanced beneficial activities of kombucha tea indicate that some changes are related to the origin of the microbial community that is present during the fermentation process [4,5,6].

Non-fermented tea such as green tea contains major polyphenolic catechins, such as epigallocatechin gallate (EGCG), epigallocatechin (EGC), epicatechin gallate (ECG), and epicatechin (EC) [7]. Black tea is considered a fully fermented form of tea since the production process creates a small particle size of the tea leaves and a greater surface area for enzymatic oxidation. During black tea fermentation, quinones react with catechins and produce new compounds; theaflavins and thearubigins [8]. In addition, catechins present in green tea are also partially converted to theaflavins [9]. Oolong tea is classified as semi-fermented tea and prepared during a limited period of oxidation. Thus, the fermentation process of oolong tea is shorter than that of black tea. Notably, oolong tea contains approximately half the amount of catechins when compared to green tea [10]. The compounds that are produced during the production processes of different types of tea markedly affect the composition and total phenolic content that are found in kombucha tea. The major polyphenolic components, catechin and epicatechin, are known to possess antioxidant activity [11]. Moreover, the antimicrobial activity of kombucha from black tea against pathogenic *Vibrio* strains was observed [12].

Although kombucha has been used for long time but scientific report on properties of kombucha has not been clarified. In this study, different biological properties of kombucha tea from various kinds of tea leaves including green, oolong, and black tea were determined for the useful properties of kombucha and application as supplementary beverage for health benefits. Hence, the aim of this study was to investigate the antioxidant and antibacterial properties of kombucha that was obtained from different types of *C. sinensis*, including green tea, oolong tea, and black tea, and the degree of toxicity it displays against the colorectal cancer cell line.

## 2. Materials and Methods

### 2.1. Starter Culture of Kombucha Tea

Tea leaves and starter culture were also provided from Tea Gallery Group (Thailand) Co., Ltd., Chiang Mai, Thailand. Kombucha tea was prepared using the same method of the industry standard. The formulation of kombucha tea production consisted of 1% of tea leaf and 10% of sucrose. The starter culture of kombucha tea was used as a consortium culture combining yeast and acetic acid bacteria. The stock culture of kombucha tea contained a total count of bacterial colony at 7.79–7.81 log CFU/mL and the yeast colony was recorded at 7.53–7.75 log CFU/mL.

### 2.2. Preparation of Kombucha Tea Obtained from Camellia Sinensis Tea Leaves

Kombucha tea was prepared using different types of tea; green, oolong, and black tea. The tea leaves were obtained from the Tea Gallery Group (Thailand) Co., Ltd. Dry tea leaves at 1.0% (*w*/*v*) were added to 500 mL of sterilize distilled water and then boiled for 15 min. Sucrose was filtered into sterilized glass bottles and 10% (*w*/*v*) sucrose was then dissolved in the hot tea. A starter culture made from the fermentation broth of the tea fungus at 10% (*v*/*v*) was inoculated into a mixture of tea and sugar, and it was further incubated at room temperature for 15 days. Subsequently, the kombucha tea was analyzed for its chemical and microbiological properties at 0, 3, 6, 9, 12, and 15 days of fermentation.

### 2.3. pH and Total Acidity Determination

The pH of kombucha tea was measured using an electronic pH meter (Denver Instruments, Bohemia, NY, USA). The total acidity of kombucha tea was measured according to the procedure of Srihari and Satyanarayana [13]. Kombucha tea was titrated with 0.1 M NaOH. The volume of the NaOH solution was calculated in terms of grams of acetic acid per liter of the sample.

### 2.4. Total Soluble Solids (ºBrix) and Alcohol Content

The total soluble solids (*ºBrix*) were measured using a refractometer (RHB-62ATC, JEDTO, Halden, Norway) and the alcohol content of the kombucha tea was measured using an Ebuliometer (Dujardin Salleron, France).

### 2.5. Microbiological Determination

The total count of yeast cells in the kombucha tea was determined using the spread plate technique on yeast malt (YM) agar with the addition of chloramphenicol. Determination of the total count of acetic acid bacteria was performed on yeast peptone mannitol (YPM) agar with amphotericin B (CAISSON, USA). The plates were incubated at 30 °C, for at least 3 days [14].

### 2.6. HPLC Analysis of Organic Acids

The predominant organic acids found in kombucha tea were determined by high performance liquid chromatography (HPLC). Six organic acids including glucuronic acid (Sigma-Aldich, Darmstadt, Germany), gluconic acid (Merck, Darmstadt, Germany), d-saccharic acid 1,4-lactone (Sigma-Aldich, Germany), acetic acid (Merck, Germany), ascorbic acid (Merck, Germany), and succinic acid (Merck, Germany) in kombucha tea were optimized for detection by isocratic HPLC systems with a conventional C18 column. The kombucha tea samples were filtered through a 0.22 µm sterile microfilter and 50 μL of the filtrate was injected into the HPLC system (Agilent technologies 1200 series, Santa Clara, CA, USA). The C-18 column (4.6 × 150 mm, 5 µm; GL Sciences, Tokyo, Japan) employing a UV detector (210 nm) was used for the analysis. Moreover, the HPLC system was controlled with a flow rate of 0.8 mL per minute and a running time of 40 min at 25 °C. Six organic acids in kombucha tea were separated by 20 mM KH_2_PO_4_ elution buffer at pH 2.4 and the standards of organic acids were used for comparison with kombucha tea. The peak area of each organic acid was calculated by Agilent ChemStation level-5 program, and then standard graphs of each organic acid were generated. Thus, the content of each organic acid in kombucha tea was calculated from the standard graph of each organic acid.

### 2.7. DPPH Radical Scavenging Assay of Kombucha Tea

The radical scavenging activity of kombucha tea was determined against DPPH free radical content [15]. The kombucha tea was diluted with methanol by 10-fold serial dilution. Each concentration of the kombucha tea (0.5 mL) was incubated with 1.5 mL of 0.1 mM DPPH solution (Sigma-Aldich, Germany). Absorbance at 517 nm was measured by spectrophotometer (Genesys 20, Thermo Scientific, Dreieich, Germany) after the solution mixture was kept in the dark at room temperature for 20 min. Methanol was used as a blank solution, while DPPH without kombucha tea was used as a control. The absorbance of the DPPH solution A1, and the absorbance of kombucha tea mixed with the DPPH solution A2, were measured. The percentage of DPPH free radical inhibition was calculated as follows: percentage inhibition = {(A1–A2)/A1} × 100.

The antioxidant activity of kombucha tea was assessed by comparing the samples to standard gallic acid and was expressed as milligrams of gallic acid per milliliter of kombucha tea (mg GAE/mL kombucha tea).

### 2.8. Total Phenolic Content of Kombucha Tea

Total phenolic content of kombucha tea was determined by Folin-Ciocalteu reagent [16]. Kombucha tea (250 μL) was mixed with 125 μL of 50% Folin-Ciocalteu reagent (Merck, Germany) and 250 μL of 95% ethanol. The mixture was incubated in the dark at room temperature for 5 min. Subsequently, 250 μL of 5% sodium carbonate was then added, and the mixture was incubated in the dark at room temperature for 60 min. Blue molybdenum–tungsten complex was formed and detected at 725 nm by spectrophotometer (Genesys 20, Thermo Scientific, Germany). Total phenolic content was calculated by comparing the substance to standard gallic acid and was expressed as milligrams of gallic acid per milliliter of kombucha tea (mg GAE/mL kombucha tea).

### 2.9. Pathogenic Enteric Bacteria Used in the Study

The pathogenic enteric bacteria that were used for the antimicrobial activity test included *E. coli* O157:H7 DMST 12743, *Shigella dysenteriae* DMST 1511 and *Salmonella* Typhi DMST 22842. *Escherichia coli* and *Vibrio cholera* were kindly obtained from the Microbiology Section, Department of Medical Technology, Faculty of Associated Medical Science, Chiang Mai University, Chiang Mai, Thailand. The bacterial strains were stored in glycerol stock at −20 °C and then grown on Mueller–Hinton (MH) agar (Difco™, Detroit, MI, USA) plates at 37 °C for 18–24 h.

### 2.10. Antimicrobial Activity of Kombucha Tea

A single colony of the tested bacteria; *Escherichia coli*, *E. coli* O157:H7, *Shigella dysenteriae*, *Salmonella* Typhi, and *Vibrio cholera*, was transferred into Mueller–Hinton (MH) broth (Difco™, USA) and incubated at 37 °C for 18–24 h. The antimicrobial activity of kombucha tea after 15 days of fermentation was investigated using the agar well diffusion method. The turbidity of the bacterial culture was adjusted to McFarland standard no. 0.5 and swabbed on Mueller–Hinton (MH) agar (Difco™, USA). Wells of 10 mm in diameter were prepared on the agar plate with a sterile cork borer. Kombucha samples were sterilized by filtering them through a sterile microfilter (0.22 μm pore size), and they were then transferred into the wells in the agar plates. The plates were further incubated at 37 °C for 18–24 h. The zones of bacterial growth inhibition were then determined [17,18].

The antimicrobial activity of kombucha tea was compared to the non-fermented tea, and acetic acid was recorded at the same concentration of the kombucha tea after 15 days of fermentation. Moreover, kombucha tea was neutralized at pH 7.0 by adjusting the pH with 1 M HCl or 1 M NaOH. Heat denatured kombucha tea was prepared by treatment at 100 °C for 20 min and at 121.5 °C, at 15 pounds per square inch for 15 min by being autoclaved. After treatment, the kombucha tea was then sterilized by filtration and tested for its antimicrobial activity, as has been described previously.

### 2.11. Cytotoxicity Test of Kombucha Tea on Human Colorectal Carcinoma (Caco-2) and NIH/3T3 Cells

The cytotoxicities of kombucha tea were tested using MTT assay. NIH/3T3 cells were used as normal cell control. Human colorectal carcinoma (Caco-2) and NIH/3T3 cells were cultured in Dulbecco’s modified Eagle medium (DMEM; Gibco, Grand Island, NY, USA) that had been supplemented with 10% (*v*/*v*) heat-inactivated fetal bovine serum (HyClone^TM^, Pittsburgh, PA, USA), 100 Units/mL penicillin and 100 μg/mL streptomycin (CAISSON, Smithfield, UT, USA). After incubation at 37 °C in a 5% CO_2_ incubator (SHEL LAB, USA), the cells were washed twice with phosphate buffer saline (PBS, pH 7.4) and trypsinized with 0.05% (*v*/*v*) trypsin-EDTA solution (CAISSON, USA). The Caco-2 and NIH/3T3 cells were plated in 96-well plates and incubated at 37 °C in a 5% CO_2_ incubator for 24 h. After incubation, each concentration of kombucha tea was then added. The plates were incubated at 37 °C in a 5% CO_2_ incubator for 48 h. The MTT solution (Bio Basic Inc., Amherst, NY, USA) was then added and the solution was incubated for 4 h. Finally, blue formazan crystals were dissolved with dimethyl sulfoxide and the absorbance was measured at 540 and 630 nm by micro plate reader (EZ Read 2000, Biochrom, Cambridge, UK). The percentage of cell viability was calculated by comparing the relevant values to the cell control [19].

### 2.12. Statistical Analysis

All experiments were performed in three independent treatments. All data acquired from the treatments and the control groups were compared and analyzed and are presented as mean ± SD using *t*-test and ANOVA analysis.

## 3. Results

### 3.1. Appearance of Kombucha Tea during Fermentation

**Figure 1:**
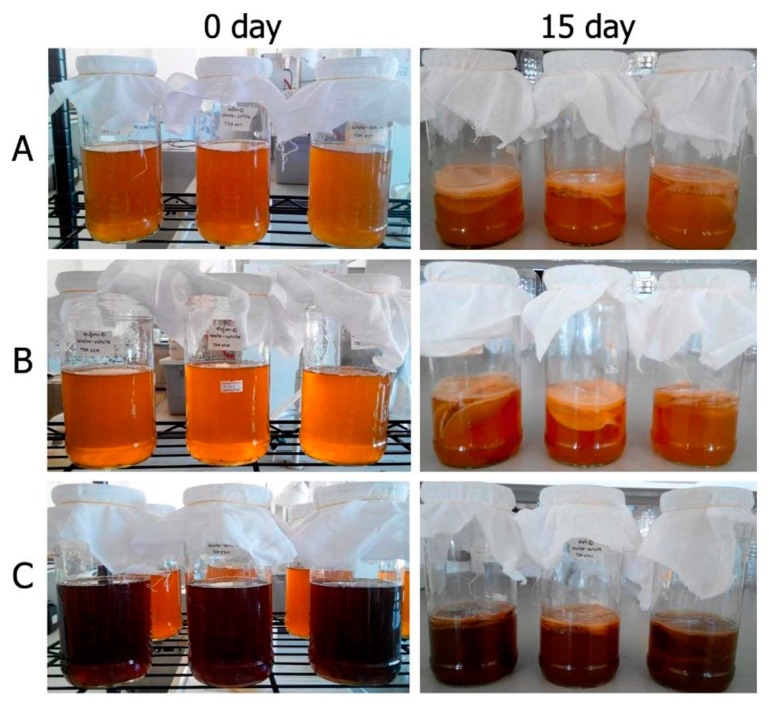
Appearance of kombucha prepared from green tea (**A**), oolong tea (**B**), and black tea (**C**) at the beginning of the fermentation process and after 15 days of fermentation.

Kombucha tea was prepared from green, oolong, and black teas using 1% (*w*/*v*) tea leaves and 10% (*v*/*v*) fermentation broth in the preparation of tea fungus as a starter culture. After 15 days of fermentation, the kombucha tea prepared from black tea appeared as a dark-brown color, while the kombucha tea that was prepared from the green tea and oolong tea displayed a brown color. During the fermentation period of kombucha preparation over 3–15 days, a thin film appeared on top of the culture medium, which was identified as the cellulose produced by the acetic acid bacteria (Figure 1).

### 3.2. Microbial and Chemical Changes that Occur During Kombucha Tea Fermentation

**Figure 2:**
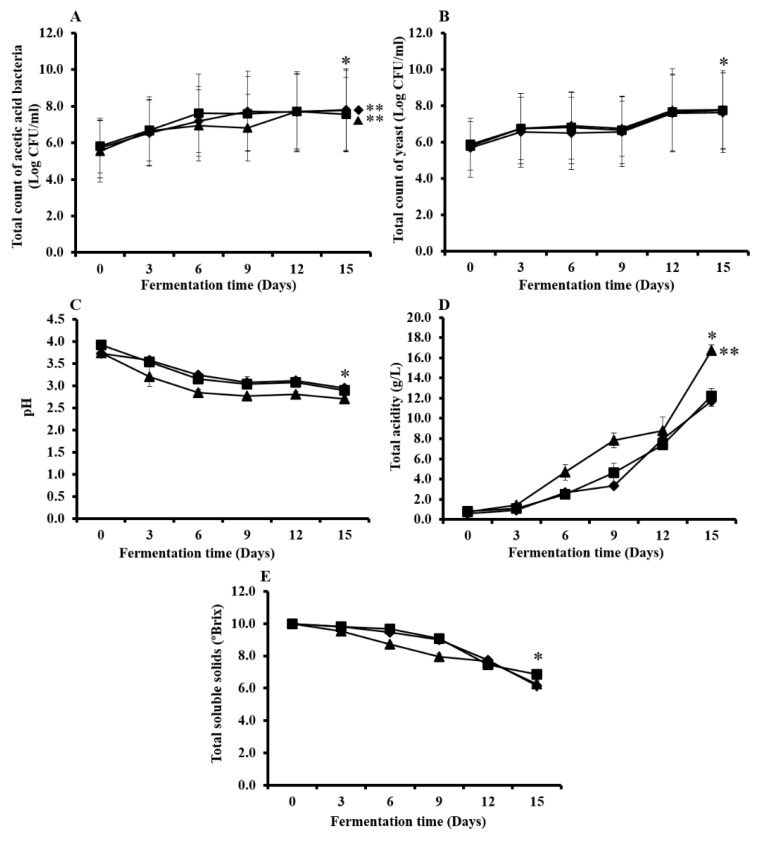
Alteration of the total count of acetic acid bacteria (**A**), total count of yeast cells (**B**), pH (**C**), total acidity (**D**), and total soluble solids (**E**) during fermentation of kombucha prepared from green tea (◆), oolong tea (■), and black tea (▲). * The values were significantly different between the beginning of the fermentation and the end of 15 days of the fermentation period (*p* < 0.05). ** The values were significantly different for each type of kombucha tea at the end of 15 days of fermentation (*p* < 0.05). The results are presented as mean ± SD of three independent experiments.

Microorganisms in kombucha tea utilize a carbon source and begin to produce cellulose which appears as a thin layer on top of the culture medium. The different types of tea; green, oolong, and black tea, that were used in this study did not affect the growth of the tea fungus. The total counts of bacterial and yeast cells during kombucha tea fermentation are shown in Figure 2. After inoculation of the 10% (*v*/*v*) starter culture, total counts of acetic acid bacterial colonies at the beginning of the kombucha tea fermentation process for green, oolong, and black teas were 5.72, 5.81, and 5.54 log CFU/mL, respectively. Moreover, the total counts of yeast cells at the beginning of kombucha tea fermentation were 5.68, 5.89, and 5.79 log CFU/mL in the kombucha tea prepared from green, oolong, and black teas, respectively. After increasing the fermentation time, the number of bacterial and yeast cells were found to be significantly higher than when observed at the beginning of the fermentation period (Figure 2A,B). After 15 days of fermentation, a total count of 7.80 log CFU/mL was found in the kombucha that had been prepared from green tea and black tea. The acetic acid bacterial colonies were significantly higher in the kombucha prepared from green and black teas than in the kombucha prepared from oolong tea (7.54 log CFU/mL). In addition, the total counts of the yeast cells in the kombucha tea prepared from green tea (7.64 log CFU/mL), oolong tea (7.74 log CFU/mL) and black tea (7.78 log CFU/mL) were not significantly different in each type of kombucha tea.

The blank control without acetic acid and yeast was performed. However, after incubation for 2–3 days the contamination from other microorganisms was presented because the blank control showed pH around 4.57–5.25. However, pH of 3.73–3.92 was determined after inoculation of starter culture at 0 day of fermentation time (Figure 2C). This acidic condition of kombucha tea inhibited other contaminated microorganisms in kombucha tea.

The alteration of pH values during kombucha tea fermentation with significantly different initial pH values is shown in Figure 2C. At the end of the 15-day fermentation period, the pH value of kombucha tea that had been prepared from black tea was the lowest at a pH value of 2.70. Kombucha that was prepared from green tea and oolong tea revealed pH values of 2.94 and 2.89, respectively. On the other hand, changes in titratable acidity that occurred during the fermentation process were significantly increased, which indicated a concentration of these organic acids (Figure 2D). The total acidity of the kombucha prepared from black tea was significantly higher (16.75 g/L) than that of the kombucha prepared from oolong (12.24 g/L) and green teas (11.72 g/L). In contrast, total soluble solids of kombucha prepared from green, oolong and black tea were significantly decreased from 10 *°Brix* to 6 *°Brix* at 15 days of fermentation (Figure 2E). In addition, no alcohol content was detected during the process of kombucha tea fermentation.

### 3.3. Organic Acids in Kombucha Tea

**Figure 3:**
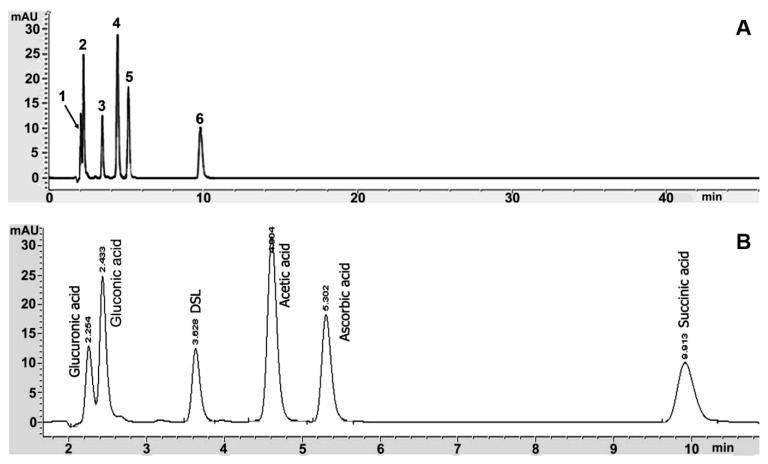
HPLC chromatograms of running time 40 min (**A**) and the six organic acids (**B**) are presented as standards; glucuronic acid (Peak 1), gluconic acid (Peak 2), D-saccharic acid 1,4-lactone (DSL) (Peak 3), acetic acid (Peak 4), ascorbic acid (Peak 5), and succinic acid (Peak 6).

The organic acids in kombucha tea were analyzed by HPLC assay. The HPLC system was optimized for the detection of several organic acids in kombucha tea with a conventional C18 column. HPLC conditions were optimized and 20 mM KH_2_PO_4_ with a pH value of 2.4 in the isocratic elution buffer was used with a 210 nm UV detector. Six organic acids including glucuronic acid, gluconic acid, D-saccharic acid 1,4-lactone (DSL), acetic acid, ascorbic acid, and succinic acid were clearly separated. Chromatograms of six standard organic acids, including glucuronic acid, gluconic acid, DSL, acetic acid, ascorbic acid, and succinic acid, were eluted at different retention times (Figure 3).

**Figure 4:**
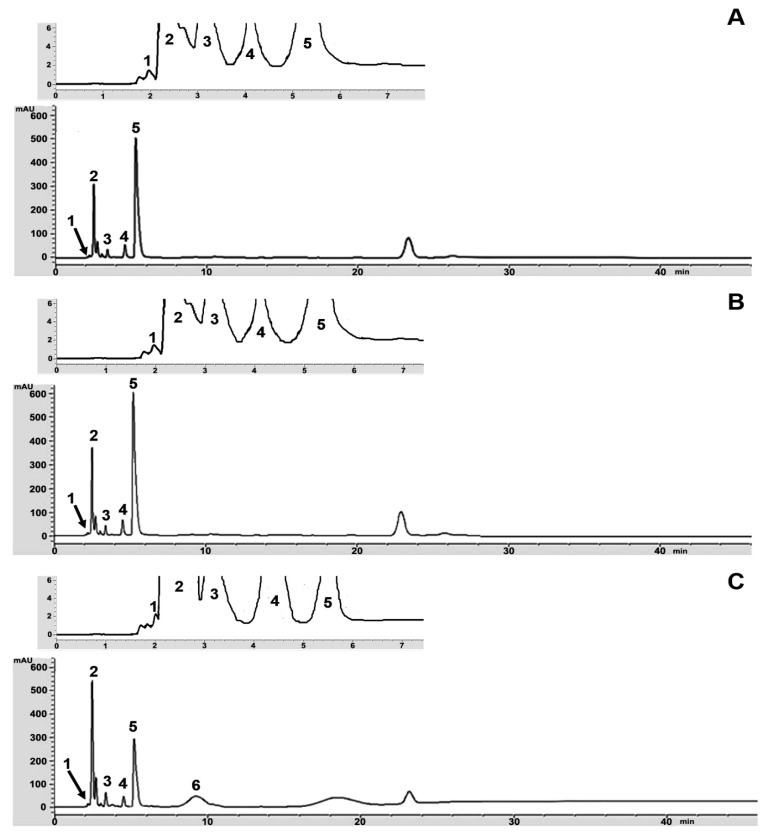
HPLC chromatograms of kombucha prepared from green tea (**A**), oolong tea (**B**), and black tea (**C**) at 15 days of fermentation. Peak (**1**)—glucuronic acid; Peak (**2**)—gluconic acid; Peak (**3**)—D-saccharic acid 1,4-lactone (DSL); Peak (**4**)—acetic acid; Peak (**5**)—ascorbic acid; Peak (**6**)—succinic acid, respectively.

After 15 days of kombucha fermentation, the amounts of each type of organic acid that were present in the kombucha prepared from green, oolong, and black teas were analyzed by HPLC. The chromatograms and production values of organic acids at 15 days of fermentation are shown in Figure 4 and Table 1. The highest content of gluconic acid was found in the kombucha that was prepared from green, oolong, and black teas, followed by acetic acid, DSL, ascorbic acid, and glucuronic acid. In contrast, succinic acid was detected in the kombucha that had been prepared from black tea, but it was not detected in the kombucha prepared from green tea and oolong tea. Therefore, kombucha prepared from black tea revealed the highest amounts of organic acid content: glucuronic acid (1.58 g/L), gluconic acid (70.11 g/L), DSL (5.23 g/L), ascorbic acid (0.70 g/L), acetic acid (11.15 g/L), and succinic acid (3.05 g/L), when compared to both oolong tea and green tea.

### 3.4. DPPH Scavenging Ability and Total Phenolic Content during Kombucha Fermentation

**Figure 5:**
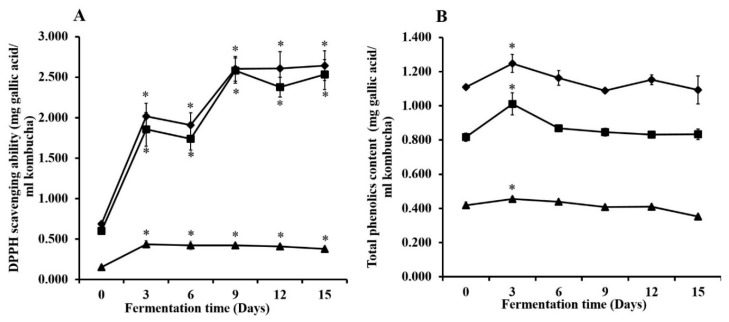
DPPH scavenging ability (**A**) and total phenolic content (**B**) during fermentation of kombucha prepared from green tea (◆), oolong tea (■), and black tea (▲). * The values were significantly different between the beginning of the fermentation and on each day of the fermentation period (*p* < 0.05). The results are presented as mean ± SD of three independent experiments.

The DPPH scavenging ability of kombucha prepared from green, oolong, and black tea significantly increased during the fermentation process to 2.642, 2.582, and 0.435 mg GAE/ml kombucha tea, respectively (Figure 5). The maximum increase of DPPH scavenging activity was presented after 15 days in the kombucha that was prepared from green tea. Kombucha prepared from oolong tea showed the highest DPPH scavenging activity after 9 days of fermentation and was remarkably stable after 12–15 days of fermentation. Moreover, the DPPH scavenging ability of the kombucha prepared from black tea was at the highest incremental value after 3 days of the fermentation process.

Total phenolic content is shown in Figure 5. After fermentation for three days, the kombucha that was prepared from green, oolong, and black teas revealed maximum levels of total phenolic contents at 1.248, 1.011, and 0.455 mg GAE/mL kombucha tea, respectively. After 3–6 days of fermentation, the amounts of phenolic compounds decreased and then appeared to be stable at 15 days of the fermentation process. Thus, kombucha prepared from green tea revealed the highest amounts of antioxidant activity and total phenolic content.

### 3.5. Total Acidity and Antibacterial Activity of Kombucha Tea

The antibacterial activities of the kombucha tea that was fermented under different conditions were investigated against pathogenic enteric bacteria. The fermented tea was tested at 15 days in terms of acidity and then after neutralization (pH 7.0). Moreover, the samples were heated to analyze the thermostability of the active components. Kombucha tea prepared from green, oolong, and black tea showed antibacterial activity against all tested enteric bacteria; *Escherichia coli*, *E. coli* O157: H7 DMST 12743, *Shigella dysenteriae* DMST 1511, *Salmonella* Typhi DMST 22842, and *Vibrio cholerae*. The diameter of the inhibition zones of the kombucha prepared from green tea ranged from 20.0 ± 0.0–24.7 ± 0.6 mm. The diameter of the inhibition zones of kombucha prepared from oolong tea ranged from 19.3 ± 0.6–24.7 ± 0.6 mm and the diameter of the inhibition zones of black tea ranged from 20.0 ± 0.0–21.3 ± 0.6 mm. The antibacterial activity of each type of kombucha tea was similar to that of the acetic acid at the same total acidity content of each type of kombucha tea. In this study, gentamicin (1 mg/mL) was used as a positive control and presented the diameters of the inhibition zones ranged from 19.3 ± 0.6–23.3 ± 0.6 mm. after testing with *E. coli*, *E. coli* O157:H7, *S. dysenteriae*, *S.* Typhi, and *V. cholera* (Table 2).

On the other hand, the antibacterial activity of kombucha after neutralization to pH 7.0 did not reveal any inhibition of all of the tested enteric bacteria. Kombucha tea was also treated by being boiled (100 °C for 20 min) and autoclaved (121.5 °C for 15 min) to determine the activity of the thermostability of its components. The total acidity of the kombucha tea prepared from green, oolong, and black teas were titrated after thermal treatments, while the total acidity of kombucha tea prepared from green, oolong, and black teas after treatment by boiling was 10.93, 11.61, and 15.67 g/L, respectively. In addition, the total acidity of kombucha tea from green, oolong and black teas after treatment by being autoclaved was 8.12, 9.16, and 12.16 g/L, respectively. Thus, the total acidity level of kombucha tea after treatment by boiling was maintained since the total acidity was significantly higher than that of the kombucha tea that was treated by being autoclaved. The results indicated that the antibacterial activity of kombucha tea after treatment by boiling revealed significantly higher inhibitory activity on the tested enteric bacteria than in the kombucha tea that had been treated by being autoclaved (Table 2).

### 3.6. Cytotoxicity Test of Kombucha Tea Against Caco-2 and NIH/3T3 Cells

The inhibition of the kombucha in different types of tea leaves was investigated against Caco-2 cancer cells and NIH/3T3 cells by MTT assay (Table 3). Kombucha tea prepared from green, oolong and black teas at 15 days of fermentation inhibited Caco-2 cancer cells with 50% inhibitory concentrations (IC_50_) of 2.603%, 1.899%, and 6.077%, respectively. Moreover, Kombucha from green, oolong and black teas inhibited NIH/3T3 cells with 50% inhibitory concentrations (IC_50_) of 2.851%, 1.922%, and 6.697%, respectively.

The effect of kombucha tea on cancer cells showed cytotoxicity values higher than cytotoxicity on normal cells. However, kombucha tea prepared from green tea and black tea significantly showed toxicity against cancer cells when compared to normal cells. Moreover, cytotoxicity of kombucha prepared from green tea and black tea after boiling (100 °C for 20 min) also showed significantly toxicity against cancer cells.

Moreover, all unfermented tea containing 1% (*w*/*v*) of tea leaf-infusion also displayed toxicity toward Caco-2 cancer cells. In contrast, kombucha that was neutralized with 1 M NaOH at a pH value of 7.0 revealed levels of toxicity on Caco-2 cells that were lower than those in the heat-denatured kombucha. Acetic acid was used for acidic activity control and was prepared with the same total acidity content of kombucha for each type of tea. After treatment of Caco-2 cancer cells with acetic acid, IC_50_ values of the kombucha prepared from green, oolong, and black teas were 33.155%, 12.610%, and 8.720%, respectively. Thus, the toxicity of the acetic acid on cancer cells was lower than in the fermented kombucha that had been prepared from each type of tea leaves.

## 4. Discussion

Kombucha tea was prepared from green, oolong, and black teas. The microbial community and chemical properties presented during kombucha fermentation were demonstrated in this study. In this study, the starter culture of kombucha tea was used as a symbiosis culture of acetic acid bacteria including *Acetobacter xylinum* and yeast cells. Therefore, cellulose production from *A. xylinum* was present on the surface of the kombucha tea during 3–15 days of fermentation. The production of the cellulose of this species is important for kombucha tea fermentation since a floating cellulose pellicle provides benefits to microorganisms by enhancement of the association between the bacteria and yeast cells and allows for the exposure of the microorganisms to atmospheric oxygen [20]. Moreover, caffeine and other related xanthines that are found in tea showed the ability to stimulate cellulose production by way of the bacteria that is present [3].

Normally, kombucha tea was completely fermented when fermentation time was 12–21 days and the inoculation size of starter culture was 5–10%. Thus, a period of kombucha fermentation of 15 days was selected in this study since the highest level of acidity was demonstrated during this fermentation process [11,21,22,23]. Moreover, yeast cells are known to convert sucrose into glucose and fructose by invertase enzymes. Additionally, ethanol was also produced. Glucose in kombucha was further utilized by acetic acid bacteria to produce gluconic acid, whereas ethanol was used to produce acetic acid. The different types of tea: green, oolong, and black tea that were used in this study did not affect the growth of the tea fungus. After increasing the fermentation time, the total counts of acetic acid bacterial and yeast cells were found to be significantly higher than were observed at the beginning of the fermentation period. After 15 days of fermentation, the total counts of acetic acid bacteria in the kombucha prepared from green tea and black tea were significantly higher than in the kombucha prepared from oolong tea. Additionally, the total counts of yeast cells in each kombucha tea were not significantly different. Therefore, the increasing amounts of bacteria present in kombucha during the fermentation process is dependent upon the different types of tea that are used as a substrate [3].

The pH values of the kombucha prepared from green, oolong, and black teas decreased due to the production of organic acids during the fermentation process. The lowest pH value was recorded in the kombucha that had been prepared from black tea, followed by that of oolong tea and green tea. The total acidity of kombucha prepared from black tea showed the highest total amount of acids, which related to the low pH value of the kombucha. Moreover, microorganisms were able to use sucrose as the major substrate to produce many organic acids that resulted in the decrease of total soluble solids during fermentation of each kombucha tea. These different values of pH and total acidity correspond to the different types of tea used and affected the degree of organic acid production. Notably, green tea is considered a non-fermented type of tea, whereas black tea and oolong tea are classified as fully fermented tea and semi-fermented tea, respectively. The process of tea preparation resulted in different oxidation levels of catechins. Green tea was found to possess the highest total phenolic content, and black tea showed the highest content of polyphenol oxidation products after the fermentation process [24].

Moreover, this study has enabled researches to optimize new conditions of HPLC for detection of several organic acids including glucuronic, gluconic, DSL, ascorbic, acetic, and succinic acid in kombucha tea. All organic acids were clearly separated by isocratic elution buffer using the C18 conventional column. Moreover, an elution buffer of 20 mM KH_2_PO_4_, pH 2.4, and a UV detector of 210 nm were suitable for the separation of each organic acid. Low molecular weight organic acids with high levels of polarity produced the greatest level of separation using mobile phase 20 mM KH_2_PO_4_ as an elution buffer. In addition, a low pH buffer was used to ensure that all acidic groups were protonated, which allowed for protection of the change from organic acids to the neutral form, thus allowing the best interaction between the organic acids and the C18 stationary phase [25]. The HPLC result revealed that gluconic acid and acetic acid were identified as the major organic acids in the kombucha tea that was prepared via the fermentation of green, oolong, and black tea for a period of 15 days.

In addition, kombucha prepared from black tea presented higher values of organic acid content in terms of glucuronic, gluconic, DSL, ascorbic, acetic, and succinic acid, when compared to oolong tea and green tea. The highest level of organic acid content in kombucha tea was found to be gluconic acid. The glucuronic acid present in kombucha has been associated with a number of benefits to the liver. Glucuronic acid plays an important role in liver detoxification and in the process associated with the excretion of exogenous chemicals known as glucuronidation [26].

The DPPH scavenging abilities of the kombucha prepared from green, oolong, and black teas significantly increased from the beginning of fermentation. During kombucha fermentation, many compounds with radical scavenging properties were obtained from the tea leaves and were considered by-products of the metabolic pathway of microorganisms. Catechins belong to polyphenols in green tea and they display high levels of antioxidant properties. Catechins also have the ability to scavenge free radicals and reactive oxygen species [13]. Notably, the increase of antioxidant potential against DPPH radicals from kombucha tea significantly reduced oxidative injuries in rats [27]. Moreover, kombucha tea prepared from green, oolong, and black teas showed significantly high amounts of total phenolic contents on day 3 of fermentation. After 3–6 days of kombucha tea fermentation, the amounts of phenolic compounds could maintain stability and then continued to be stable during 15 days of the fermentation process. The kinetics of microorganisms in kombucha fermentation increased around 3 days after beginning inoculation, which might be the reason for the enhancement of the phenolic compounds [28]. Many enzymes are produced during kombucha fermentation, such as phytase, α-galactosidase, and tannase, which are all related to the degradation of complex polyphenols to small molecules and are known to cause an increase in total phenolic compounds [29].

The study of kombucha prepared from Chinese black tea, Chinese green tea, Chinese oolong tea and Sri Lankan black tea also yielded the highest amounts of phenolic compounds on day 1 of fermentation. Notably, the phenolic compounds maintained a level of stability throughout 7 days of the kombucha fermentation period. Additionally, individual polyphenol contents yielded variations in terms of quantity in each type of tea [30]. The enzymes that were liberated from the bacteria and yeast in the tea fungus consortium degraded the complex of phenolic compounds in the tea, and the degradation was increased in the acidic environment of the fermentation process [21].

Moreover, kombucha contains other antioxidant substances, such as ascorbic acid and DSL, which were shown to be present in high levels in the kombucha that had been prepared from black tea. DSL, a derivative of D-glucaric acid, demonstrated detoxification, anticarcinogenic, and cholesterol-reduction properties [5,31,32,33]. DSL has been found to reduce ameliorate alloxan-induced type 1 diabetes by inhibiting the apoptotic death of pancreatic β-cells [5]. Moreover, DSL also revealed the greatest benefit in terms of anti-oxidative properties and displayed the ability to decrease oxidative damage to certain cellular biomolecules, such as on the lipids and proteins found in human blood platelets [34]. In addition, kombucha prepared from green tea revealed the highest level of phenolic content. An increase in the total phenolic content during kombucha fermentation that occurred from the ability of the bacteria and yeast was found to liberate enzymes, such as phytase, which could break down the cellulosic backbone of the tea leaves to release polyphenol compounds [21].

In this study, the kombucha prepared from green, oolong and black teas efficiently inhibited all tested pathogenic enteric bacteria: *Escherichia coli*, *E. coli* O157:H7 DMST 12743, *Shigella dysenteriae* DMST 1511, *Salmonella* Typhi DMST 22842, and *Vibrio cholerae*. The strongest antibacterial activity of kombucha tea was related to the presence of organic acid, such as the acetic acid found in kombucha tea. This antibacterial activity displayed a significant level of inhibitory activity against all tested bacteria. The studies by Dibner and Buttin [35] reported that organic acids displayed antimicrobial activity and also displayed an inhibitory effect against acid-intolerant species such as *E. coli*, *Salmonella* sp., and *Campylobacter* sp. that had been obtained from the guts of piglets. Weak organic acids, such as acetic acid and benzoic acid, were reported to show antimicrobial activity since the organic acid molecules could induce cytoplasmic acidification and destroy bacterial cells [36].

Kombucha tea displayed a remarkable level of antimicrobial activity against a broad range of microorganisms, which have demonstrated an ability to inhibit the growth of pathogens such as *Helicobacter pylori*, *Escherichia coli*, *Entamoeba cloacae*, *Pseudomonas aeruginosa*, *Staphylococcus aureus*, *S. epidermidis*, *Agrobacterium tumefaciens*, *Bacillus cereus*, *Aeromonas hydrophila*, *Salmonella typhimurium*, *S. enteritidis*, *Shigella sonnei*, *Leuconostoc monocytogenes*, *Yersinia enterocolitica*, *Campylobacter jejuni*, and *Candida albicans* [3,37]. Both acetic acid and catechins are known to inhibit a range of Gram-positive and Gram-negative microorganisms [2]. Therefore, kombucha tea has been recognized as containing active compound substances that could inhibit bacterial pathogens [23,38]. Battikh et al. [39] reported that kombucha prepared from green tea could inhibit *S. epidermidis*, *S. aureus*, *Micrococcus luteus*, *S. typhimurium*, *E. coli*, *Listeria monocytogenes*, and *P. aeruginosa* with diameters ranging from 12 to 22 mm, while kombucha prepared from black tea showed inhibition zones against these bacteria ranging from 10.5 to 19 mm. Furthermore, other studies showed that antibacterial activities did not exclusively from acetic acid or other organic acids such as citric acid, lactic acid, malic acid, and pyruvic acid [40] but possibly from other biologically active components such as bacteriocins, proteins, enzymes, and tea-derived phenolic compounds as well as tannins originally present in the tea broth that could have been involved as antimicrobial substances [37,38].

In contrast, unfermented tea did not display any antimicrobial activity against the tested microorganisms. This probably occurred because of the low concentrations of tea broth (1%, *w*/*v*) and polyphenol levels. Thus, unfermented tea did not display any inhibitory effects against the tested microorganisms [41]. In this study, neutralized kombucha did not display any inhibitory activity on all tested bacteria. Therefore, the use of the kombucha tea will provide natural organic acids for health benefits. The antibacterial activity of kombucha occurred from the acidity present in the kombucha tea. The same result was obtained from the previous studies of Cetojevic-Simin et al. [42], all of whom reported that neutralized kombucha prepared from black tea could not inhibit pathogenic bacteria. 

In addition, heat-denatured kombucha tea prepared after boiling (100 °C, 20 min) and autoclaving (121.5 °C for 15 min) inhibited all tested pathogenic enteric bacteria. These results confirm that the antimicrobial components in the kombucha are thermostable. Moreover, heat-denatured kombucha tea that was achieved by boiling could significantly inhibit bacteria more effectively than the heat-denatured kombucha tea that had been autoclaved. The acidity of kombucha tea treated by boiling was maintained and the amount of total acidity was significantly higher than in the kombucha tea that was treated by autoclave. Moreover, the amount of total acidity present between heat-denatured kombucha tea by boiling and kombucha tea without any treatment was not significantly different. In contrast, the total acidity of kombucha tea that was treated by autoclave significantly decreased the amount of acidity when compared to kombucha tea without any treatment. This phenomenon indicated that the acidity activity of kombucha tea was reduced at high temperature by being autoclaved. This is particularly noteworthy with regard to the pasteurization of kombucha tea beverages that were prepared to preserve the potential antibacterial agents that are present in kombucha tea. Moreover, other components, such as catechins, and the antioxidant activities recorded after the autoclaving of these tea drinks at 120 °C for 20 min also decreased [43].

Kombucha prepared from green, oolong, and black tea were found to display effective toxicity against Caco-2 colorectal cancer cells. Interestingly, kombucha prepared from green tea and black tea showed the specific toxicity on cancer cells. Therefore, this is a new report about the cytotoxicity effect of kombucha prepared from different kinds of tea leaves on Caco-2 colorectal cancer cells. However, toxicity test of kombucha tea on different types of cancer and normal cell lines should be further studied.

The major beneficial components of green tea, such as EGCG, restrained carcinogenesis in a variety of tissues through the inhibition of mitogen-activated protein kinases, growth factor-related cell signaling, activation of activator protein 1 and nuclear factor-B, topoisomerase I, and matrix metalloproteinases along with other potential targets [44]. Previous reports have shown that kombucha prepared from black tea contained dimethyl 2-(2-hydroxy-2-methoxypropylidene) malonate and vitexin that caused certain cytotoxic effects on 786-O (human renal carcinoma) and U2OS (human osteosarcoma) cells by the reduction of cell invasion, cell motility, and matrix metalloproteinase activity [45,46]. Moreover, kombucha tea was found to significantly decrease the survival rate of prostate cancer cells by downregulating the expression of angiogenesis stimulators like matrix metalloproteinase, cyclooxygenase-2, interleukin-8, endothelial growth factor, and human inducible factor-1α [47]. The active substances of kombucha are associated with many of the compounds found in each type of the tea leaves and are related to the acid production that occurs from the microorganisms.

Tea polyphenols in kombucha that are present in the tea leaves and during kombucha fermentation were identified as anticancer substances. Tea polyphenols could inhibit the mechanisms of cancer formation such as gene mutation and cancer cell proliferation. Notably, tea polyphenols also induced the apoptosis of cancer cells and terminated cancer cell metastasis [48,49,50]. Both types of tea leaves that had been infused (1%, *w*/*v*) and acidified in the kombucha tea showed the ability to inhibit Caco-2 colorectal cancer cells. In 2013, a study by Zhao et al. [51] showed that fermented and unfermented specimens of Pu-erh tea and green tea could inhibit HT-29 colon cancer. Additionally, kombucha prepared from green tea and black tea could inhibit A549 human lung carcinoma cells and Hep-2 epidermoid carcinoma, while kombucha prepared from black tea could inhibit Hep-2 cells [23].

In this study, acetic acid in kombucha displayed toxicity against Caco-2 cancer cells. However, toxicity activities against cancer cells were also found to have occurred as a result of the presence of other organic acids such as glucuronic, gluconic, DSL, ascorbic, acetic, and succinic acid. DSL had the ability to inhibit the activity of glucuronidase enzymes, such as hydrolyzed glucuronides and produced aglycones. Aglycones are known to be toxic substances that are able to induce normal cells to become cancer cells [52]. Gluconic acid, glucuronic acid, lactic acid, and ascorbic acid are known to have the ability to reduce the occurrence of stomach cancer [53]. Notably, kombucha prepared from black tea was found to contain several organic acids. In this study, black tea revealed the lowest level of toxicity on Caco-2 cells because of the presence of other components found in the black tea, such as thearubigins and theaflavin, which were consistently degraded during kombucha fermentation. In contrast, catechins in green tea and oolong tea were not degraded during kombucha tea fermentation [22]. Theaflavins in black tea have been reported to possess activity against carcinogenesis by interfering with the signaling pathways and suppressing the transcription of certain oncoproteins [54]. Anticancer and antibacterial activity decreased when kombucha tea was neutralized at pH 7.0 by adjustments with NaOH. However, the anticancer components of kombucha tea were found to be thermostable after the tea was heated by either boiling or by being autoclaved. Especially, kombucha tea prepared from green and black tea after treatment by boiling at 100 °C for 20 min that also retained the cytotoxicity effect to cancer cells. However, kombucha tea at 15 days of fermentation revealed low pH values (2.70–2.94). Thus, kombucha tea should be prepared at a pH value of around 4.2 and should not be consume in amounts of more than 4 oz per day [55]. This scientific research study clarified that kombucha tea demonstrated the health benefits of effectively treating pathogenic enteric bacterial infection, anti-oxidation, and toxicity to colorectal cancer cells, which might help to promote the consumption of kombucha beverages among consumers.

## 5. Conclusions

In the present study, kombucha tea was fermented with a symbiotic culture of acetic acid bacteria and yeasts that produced a significant amount of organic acids. The kombucha tea prepared from different types of tea, namely green, oolong, and black tea, displayed significantly different values of pH, total acidity, total soluble solids, and organic acid content. Moreover, the HPLC system for the detection of several organic acids in kombucha tea was optimized in this study using the C18 conventional column. Isocratic elution buffer of 20 mM KH_2_PO_4_, pH 2.4 with 210 nm UV detector was used as a new adaptive HPLC condition for organic acid detection. Kombucha prepared from black tea at 15 days of fermentation showed the highest degree of organic acid content, such as with glucuronic, gluconic, DSL, ascorbic, acetic, and succinic acids. These organic acids were found to be effective against pathogenic enteric bacteria *Escherichia coli*, *E. coli* O157:H7, *Shigella dysenteriae*, *Salmonella* Typhi, and *Vibrio cholera*. They attributed to a large extent of the antibacterial effect observed to organic acids because when neutralizing the kombucha samples, antibacterial effect was not observed. Moreover, kombucha prepared from green tea and black tea demonstrated toxicity on Caco-2 colorectal cancer cells. These findings indicate the greatest potential health benefits of kombucha tea with regard to inhibiting pathogenic enteric bacteria and by promoting healthy function of the digestive system in the gastrointestinal tract. In addition, kombucha displayed antioxidant activity against DPPH radicals. Therefore, kombucha tea could be considered as a potential source of compounds presenting antioxidant activity, inhibitory activity against pathogenic enteric bacteria and selective toxicity on colorectal cancer cells.

## Figures and Tables

**Table 1:** Organic acid content of kombucha tea prepared from green tea, oolong tea, and black tea at 15 days of the fermentation process.

Kombucha	Organic Acids Content (g/L)					
	Glucuronic	Gluconic	DSL	Ascorbic	Acetic	Succinic
Green tea	1.37 ± 0.01 ^c^	41.42 ± 0.02 ^c^	3.44 ± 0.03 ^c^	0.61 ± 0.00 ^a^	10.42 ± 0.00 ^b^	ND ^d^
Oolong tea	0.07 ± 0.01 ^b^	48.75 ± 0.03 ^b^	4.02 ± 0.02 ^b^	0.60 ± 0.00 ^a^	10.48 ± 0.00 ^ab^	ND ^d^
Black tea	1.58 ± 0.01 ^a^	70.11 ± 0.01 ^a^	5.23 ± 0.01 ^a^	0.70 ± 0.00 ^a^	11.15 ± 0.00 ^a^	3.05 ± 0.01 ^a^

^a, b, c^ The data of different superscript letters (a, b, c) are presented as mean ± SD of three independent experiments and revealed significantly different values for each type of kombucha tea (*p* < 0.05). ^d^ ND: organic acid in kombucha tea was not detected.

**Table 2:** Antibacterial activity of kombucha prepared from green tea, oolong tea, and black tea against pathogenic enteric bacteria.

Type of *Camellia Sinensis*	Tested Extract	^a^ Inhibition Zone Diameter (mm) of Target Bacteria				
		*E. coli*	*E. coli* O157: H7	*S. Dysenteriae*	*S.* Typhi	*V. Cholera*
Green tea	^b^ Fermented tea	24.7 ± 0.6	24.3 ± 0.6	21.7 ± 0.6	23.7 ± 0.6	20.0 ± 0.0
	^c^ Unfermented tea	0	0	0	0	0
	^d^ Neutralized kombucha	0	0	0	0	0
	^e^ Heat-denatured Kombucha M1	25.0 ± 0.0	23.0 ± 0.0	21.0 ± 0.0	23.0 ± 0.0	19.0 ± 0.0
	^f^ Heat-denatured Kombucha M2	15.3 ± 0.6	22.3 ± 0.6	19.7 ± 0.6	20.7 ± 0.6	17.3 ± 0.6
	^g^ Acetic acid (11.72 g/L)	20.3 ± 0.6	22.0 ± 0.0	20.0 ± 0.0	21.0 ± 0.0	22.0 ± 0.0
Oolong tea	Fermented tea	23.7 ± 0.6	20.3 ± 0.6	19.3 ± 0.6	24.7 ± 0.6	20.0 ± 0
	Unfermented tea	0	0	0	0	0
	Neutralized kombucha	0	0	0	0	0
	Heat-denatured Kombucha M1	20.0 ± 0.0	19.3 ± 0.6	19.3 ± 0.6	24.0 ± 0.0	16.7 ± 0.6
	Heat-denatured Kombucha M2	15.7 ± 0.6	15.0 ± 0.0	17.0 ± 0	19.7 ± 0.6	15.7 ± 0.6
	Acetic acid (12.24 g/L)	21.0 ± 0.0	20.0 ± 0.0	21.0 ± 1.0	20.3 ± 0.6	22.0 ± 0.0
Black tea	Fermented tea	21.0 ± 0.0	21.3 ± 0.6	21.0 ± 0.0	20.0 ± 0.0	21.0 ± 0.0
	Unfermented tea	0	0	0	0	0
	Neutralized kombucha	0	0	0	0	0
	Heat-denatured Kombucha M1	20.3 ± 0.6	20.3 ± 0.6	20.0 ± 0.0	18.0 ± 0.0	20.0 ± 0.0
	Heat-denatured Kombucha M2	18.0 ± 0.0	16.3 ± 0.6	19.7 ± 0.6	17.3 ± 0.6	18.0 ± 0.0
	Acetic acid (16.75 g/L)	20.0 ± 0.0	20.0 ± 0.0	20.7 ± 0.6	21.0 ± 0.0	21.7 ± 0.6
Gentamycin (1 mg/mL)		20.3 ± 0.6	19.3 ± 0.6	23.3 ± 0.6	22.3 ± 0.6	20.0 ± 0.0

^a^ Inhibition zone diameter is presented as mean ± SD of three independent experiments. ^b^ Fermented tea: kombucha tea fermented at 15 days without any treatment. ^c^ Unfermented tea: broth culture contained only 1% of tea leaves. ^d^ Neutralized kombucha: kombucha tea neutralized with NaOH (1 M) at pH 7.0. ^e^ Heat-denatured kombucha M1: kombucha tea treated at 100 °C for 20 min. ^f^ Heat-denatured kombucha M2: kombucha tea treated at 121.5 °C for 15 min. ^g^ Acetic acid: acid prepared at the same total acidity content of kombucha for each type of tea leaf.

**Table 3:** Cytotoxicity of kombucha prepared from green, oolong, and black teas on Caco-2 colorectal cancer and NIH/3T3 cells.

Type of *Camellia Sinensis*	Tested Extract	^a^ IC_50_ (%)	
		Caco-2 Cells	NIH/3T3 Cells
Green tea	^b^ Fermented tea	2.603 ± 0.072 *	2.851 ± 0.052 *
	^c^ Unfermented tea	3.661 ± 2.228	3.819 ± 0.122
	^d^ Neutralized kombucha	8.621 ± 0.685	8.915 ± 0.715
	^e^ Heat-denatured kombucha M1	1.758 ± 0.065 *	1.916 ± 0.011*
	^f^ Heat-denatured kombucha M2	1.309 ± 0.117	1.518 ± 1.202
	^g^ Acetic acid (11.72 g/L)	33.155 ± 2.834	35.206 ± 0.625
Oolong tea	Fermented tea	1.899 ± 0.242	1.922 ± 0.186
	Unfermented tea	2.097 ± 0.305	2.519 ± 0.024
	Neutralized kombucha	4.194 ± 0.081	4.671 ± 1.205
	Heat-denatured kombucha M1	1.179 ± 0.041	1.219 ± 0.012
	Heat-denatured kombucha M2	1.515 ± 0.138	1.641 ± 0.220
	Acetic acid (12.24 g/L)	12.610 ± 0.341	12.817 ± 0.452
Black tea	Fermented tea	6.077 ± 0.222 *	6.697 ± 0.030 *
	Unfermented tea	6.082 ± 0.191	6.244 ± 0.659
	Neutralized kombucha	16.339 ± 0.833	17.066 ± 1.568
	Heat-denatured kombucha M1	6.083 ± 0.053 *	6.702 ± 0.156 *
	Heat-denatured kombucha M2	5.699 ± 0.008	5.819 ± 0.209
	Acetic acid (16.75 g/L)	8.720 ± 0.047	8.810 ± 0.142

^a^ Results are presented as mean ± SD of three independent experiments. ^b^ Fermented tea: kombucha tea fermented at 15 days without any treatment. ^c^ Unfermented tea: broth culture contained only 1% of tea leaves. ^d^ Neutralized kombucha: kombucha tea neutralized with NaOH (1 M) at pH 7.0. ^e^ Heat-denatured kombucha M1: kombucha tea treated at 100 °C for 20 min. ^f^ Heat-denatured kombucha M2: kombucha tea treated at 121.5 °C for 15 min. ^g^ Acetic acid: acid prepared at the same total acidity content of kombucha for each type of tea leaf. * The values were significantly different between the cytotoxicity of Caco-2 cells and NIH/3T3 cells (*p* < 0.05).
